# Peer review: historical evolution and reviewers’ learning

**DOI:** 10.1186/s13244-026-02302-8

**Published:** 2026-05-21

**Authors:** Maria Pilar Aparisi Gómez, Francesco Sardanelli

**Affiliations:** 1 Department of Radiology, Auckland Hospital (Te Whatu Ora), Auckland, New Zealand; 2 Department of Anatomy and Medical Imaging, Faculty of Medical and Health Sciences, Waipapa Taumata Rau, University of Auckland, Auckland, New Zealand; 3 Lega Italiana per la Lotta contro i Tumori (LILT) Milano Monza Brianza, Milano, Italy

**Keywords:** Knowledge, Methodology (research), Peer review, Radiology, Science

## Abstract

**Abstract:**

Peer review is the result of a long historical evolution. From the Greek philosophy (the metaphor of Socratic maieutics) to the Cartesian method of systematic doubt and analytical reconstruction, from the informal editorial judgments of seventeenth-century scientific societies to the institutionalized systems of the twentieth century, the idea of examination of apparent certainties became what we know as “peer review.” It is a pragmatic response to the growth and specialization of science, rooted in enduring epistemological traditions, started in Europe in 1665 with the *Journal des sçavans* and *Philosophical Transactions*. Through this process, authors are compelled to justify claims, expose underlying assumptions, and transform hypotheses into defensible knowledge. In radiology—particularly in the era of quantitative imaging, artificial intelligence, and biomarker development—peer review also functions as an educational tool. It sharpens the ability to detect methodological fragility, latent bias, and to distinguish exploratory from confirmatory research. Despite limitations such as reviewer fatigue and conservatism, peer review shapes scientific reasoning, allowing reviewers to learn science and clinical practice from authors and other reviewers. More than two thousand years ago, Lucius Annaeus Seneca wrote: *Homines etiam cum alios aliquid docent, aliquid discunt*—Even when humans teach others, they themselves learn something.

**Critical relevance statement:**

While reviewing manuscripts is a time-demanding activity, for the reviewer, it can result in a substantial improvement in scientific and clinical knowledge that can be transferred to the daily practice of radiology.

**Key Points:**

- Peer review evolved from informal judgment into a structured scientific safeguard.
- A good peer review asks questions rather than simply giving verdicts.
- Reviewing papers is one of the most powerful ways scientists learn.
- Peer review shapes not only science, but the reviewers themselves as scientists.

**Graphical Abstract:**

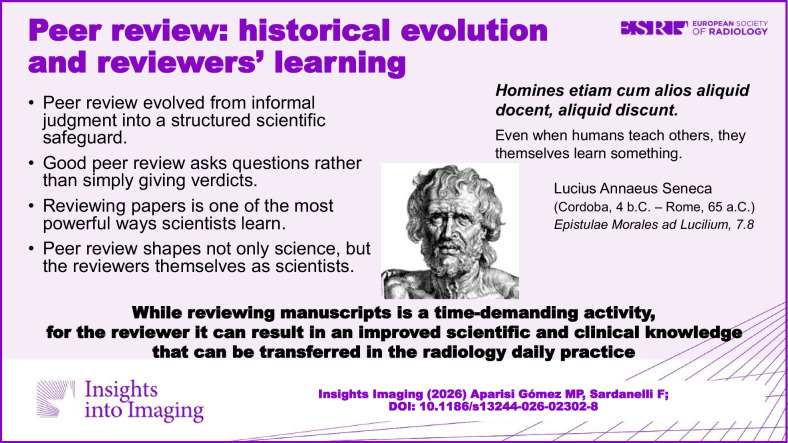

## A long historical background

The system of scientific journal peer review is so deeply embedded in modern research culture that it often appears timeless. In reality, it is a relatively recent social technology whose evolution reflects changing ideas about authority, trust, and the organization of knowledge [1].

### From Greek philosophy to Descartes

The current practice of scientific peer review is commonly described in procedural terms, such as reporting checklists and statistical thresholds. However, its underlying epistemic basis is less frequently discussed. A useful conceptual framework for understanding peer review is Socratic maieutics, the method of eliciting knowledge through structured questioning rather than simple transmission of information. When combined with the rationalist principles of the scientific method, as formulated by René Descartes, this approach provides a theoretical foundation for modern peer review [2, 3].

In Plato’s Theaetetus, Socrates describes himself as a “midwife” of ideas, claiming that he does not teach doctrines but helps others develop and refine their knowledge through questioning (Theaetetus 150b–151d) [4]. This metaphor of *maieusis* (μαιευτική), “the art of midwifery,” is more than rhetorical. This method involves the systematic examination of apparent certainties in order to identify hidden assumptions, reveal inconsistencies, and improve weak or unsupported claims. Socrates emphasized that he did not possess the truth, but worked collaboratively with others to approach it through dialogue and reasoning. In Plato’s Apology, Socrates presents this practice as a moral obligation, arguing that beliefs that are not critically examined should not be accepted (Apology 38a) [4].

Maieutics is not a form of destructive skepticism but is based on *elenchus*, a constructive mode of refutation aimed at improving the quality of knowledge. Through the requirement to define terms, justify inferences, and address counterexamples, Socratic dialogue anticipates core elements of modern methodological reasoning, long before modern philosophy of science [5].

In this framework, the validity of a claim does not depend on the authority of the speaker but on its capacity to withstand systematic questioning and to be supported by coherent argumentation and evidence.

Modern peer review reproduces this maieutic logic in a depersonalized and anonymized setting. Reviewers do not impose their own theories but instead interrogate the manuscript through standardized questions: what is being claimed, which assumptions underlie the methods, and whether the data adequately support the conclusions. These steps function as contemporary equivalents of Socratic probing, in which weak hypotheses fail under scrutiny and stronger ones are reformulated with greater precision [6]. The most valuable peer review, therefore, goes beyond error detection. It stimulates epistemic effort in the author, promoting awareness of hidden assumptions, unrecognized confounders, circular reasoning, and overinterpretation, and thereby strengthening the scientific argument [7].

The review process can be understood as a maieutic space in which scientific claims are not passively accepted but actively developed into defensible knowledge.

René Descartes provided the scientific revolution with its most influential methodological charter in the *Discours de la méthode* (1637). His four principles—accept only what is evident; divide problems into parts; progress from simple to complex; and conduct comprehensive reviews—are often taught as the grammar of modern science [8]. These principles closely parallel Socratic dialectic: including the suspension of belief (*aporia*), the decomposition of vague concepts into analyzable elements, and their reconstruction through rational inference [3].

While Socrates relied on dialogical interrogation and Descartes formalized introspective reasoning, both shared the view that knowledge arises from structured critique rather than the accumulation of facts. Effective peer review integrates these traditions by applying the Cartesian method within a Socratic-style dialogue [9].

There is also an ethical dimension. In the *Republic*, Plato links dialectic to the governance of the soul and the political organization, suggesting that intellectual rigor is inseparable from moral responsibility [4]. In scientific culture, this ethic reappears in the reviewer’s obligation to challenge constructively, to question without dogmatism, and to expose error without humiliation. A reviewer who merely rejects with no feedback is not practicing maieutics but exercising authority: (s)he is authoritarian, not authoritative. The maieutic reviewer, by contrast, provides constructive insight: creates the conditions for reflection and learning [10].

This is particularly salient in fields where complex models or high-dimensional data can mask fragile reasoning. Here, maieutic peer review serves as a cognitive safeguard against what Descartes would have called “precipitation”—the premature acceptance of results that have not been adequately decomposed or re-examined.

Socratic maieutics and the Cartesian method are not relics but complementary pillars of scientific epistemology, current in this century. The former provides the dialogical ethic of inquiry; the latter supplies its procedural architecture. Peer review stands at their intersection. When practiced as a genuine maieutic enterprise, it does more than filter manuscripts: it midwifes knowledge, transforming tentative conjectures into articulated, defensible scientific claims. Thus, every rigorous peer review reenacts a dialogue that began in the Athenian ἀγορά and was rationally codified in the seventeenth century—a reminder that science advances by disciplined questioning.

Centuries later, Lucius Annaeus Seneca (Cordoba, 4 b.C.—Rome, 65 a.C.) would highlight *Homines etiam cum alios aliquid docent, aliquid discunt*—Even when humans teach others, they themselves learn something (*Epistulae Morales ad Lucilium*, 7.8) [11].

### From *Philosophical Transactions* and *Journal des sçavans* to the twentieth century

The origins of scholarly evaluation lie in the seventeenth century, with the birth of scientific societies. When the Royal Society of London founded *Philosophical Transactions* in 1665 under the editorship of Henry Oldenburg, editorial decisions were initially personal and informal. Oldenburg consulted trusted colleagues, but there was no standardized review process. What mattered most was the reputation of the author and the editor’s own judgment. Similar practices characterized the *Journal des sçavans* in France, founded by Denis De Sallo in the same year. Early science operated within small epistemic communities in which social proximity was a substitute for formal evaluation [12–14].

During the eighteenth century, academies such as the *Académie des Sciences* in Paris began to introduce more systematic manuscript assessments. Papers submitted to the academy were read by appointed *rapporteurs*, who produced written reports for internal circulation. This marked the first institutionalization of critical evaluation, but the process remained opaque and largely advisory. Reviewers were not anonymous, and editorial authority remained concentrated within élite scientific bodies [15, 16].

The nineteenth century brought an explosion in scientific output driven by industrialization, professionalization, and the expansion of universities. Educated societies could no longer rely on informal judgment alone. Journals proliferated, disciplines fragmented, and editors increasingly depended on external experts to judge specialized work. By the late nineteenth century, journals such as *The Lancet* and *Nature* had adopted recognizable forms of external refereeing, though still without the modern norms of anonymity or standardized criteria [17, 18].

The twentieth century saw the consolidation of peer review into its modern form. After the Second World War, science became a large-scale, partly publicly funded enterprise, particularly in the United States and Europe. Governments demanded accountability for research spending, and journals faced unprecedented submission volumes. To manage this complexity and to protect scientific credibility, peer review was formalized: reviewers were selected for subject expertise, written reports became mandatory, and the anonymity of reviewers—single-blind or double-blind—was introduced to reduce bias and social pressure [2, 19, 20].

By the 1970s, peer review was widely regarded as the cornerstone of scientific legitimacy, though critics began to highlight its limitations, including conservatism, inconsistency, and susceptibility to bias [21, 22]. The late twentieth and early twenty-first centuries introduced further adaptations: focused statistical reviewers, reporting checklists, conflict-of-interest disclosures, and, more recently, open peer review and post-publication commentary enabled by digital platforms [23, 24].

Thus, scientific peer review evolved from the personal judgment of seventeenth-century editors into a complex, institutionalized system of distributed expertise. It did not arise from a single philosophical blueprint but from practical necessity: the need to manage growing volumes of knowledge while maintaining trust. Today’s peer review system, for its imperfections, remains a central mechanism through which the scientific community collectively decides what counts as reliable knowledge.

## Peer reviewing today

Today, peer reviewing is often framed as a service to journals, but in reality, it is one of the most powerful—and often under-acknowledged—forms of scientific education. What we learn when we review others’ work goes far beyond the specific manuscript in front of us.

For the radiologist, working in a field increasingly driven by quantitative imaging, artificial intelligence, and biomarker discovery, peer review is also one of the most formative acts of scientific education. It is not merely where we judge science, but where we learn how science is constructed.

We learn from the contents of what we review, but we also learn about science itself.

### Peer review and hypothesis formation

By reviewing manuscripts, we see how real scientific questions are framed under practical constraints. We observe how authors move from an observation to a testable hypothesis, how assumptions silently enter models, and how wording is shaped to survive scrutiny.

This trains us to distinguish “exploratory narrative” from “confirmatory science”—a skill that is rarely taught formally, but crucial.

By reviewing manuscripts, one quickly loses the presumption that hypotheses are always conceived a priori. In radiology, many studies originate from opportunistic observations: a texture feature that appears predictive in a pilot cohort, for example [25]. During peer review, one repeatedly encounters hypotheses that have been shaped—or reshaped—by the data: data availability, scanner limitations, or cohort heterogeneity, as examples [26, 27]. This recognition sensitizes the reviewer to the difference between hypothesis-driven and data-driven imaging science, encouraging stricter preregistration and clearer distinction between exploratory and confirmatory analyses.

Why is this important? Because, for example, the same dataset can be analyzed in many different ways—different regions of interest, thresholds, models, or endpoints. If we look at the data first and only then decide what our “hypothesis” is, we naturally end up highlighting the patterns that look significant. This phenomenon—sometimes termed *HARKing* (Hypothesizing After Results are Known) [28]—systematically inflates effect sizes, undermines reproducibility, and explains why, for example, many imaging biomarkers fail external validation.

Preregistration simply means deciding in advance what the plan is to test, it is having a protocol. Committing in advance to endpoints, inclusion criteria, segmentation strategies, and statistical models creates a clear line between what we were trying to prove and what was noticed along the way. This makes the results more trustworthy. It can then be distinguished what was tested from what was merely observed.

Exploring data is how new ideas are born. But only when those ideas are tested later, under predefined conditions, can we say they work.

### Peer review and the detection of flaws and bias

Peer review repeatedly exposes where science is weakest: in the methods. Underpowered cohorts, circular reference standards, hidden confounders, unvalidated imaging surrogates, inappropriate statistical thresholds… these are examples of situations that affect results.

In radiology, examples of these methodological flaws may translate into situations like small sample sizes for multiparametric magnetic resonance imaging analysis, scanner-specific feature drift, inconsistent segmentation strategies, inappropriate pooling of heterogeneous acquisition protocols (very typical in body composition studies based on computed tomography acquisitions), and insufficient harmonization across centers, as examples. Of note, errors may be camouflaged by technical sophistication.

A practical implication for reviewers is that over time, a reviewer develops a mental library of “failure modes.” This pattern recognition may be transformative if the reviewer is a researcher—one starts anticipating errors in one’s own work. This ultimately improves study designs.

Bias in radiological research is often hidden in post hoc subgroup rationalization, selective exclusion criteria, and language “exaggeration” unsupported by effect sizes. Sometimes it is embedded in choices: excluding “difficult” cases, redefining endpoints post hoc, or relabeling borderline associations as clinically meaningful… Reviewing teaches that expressions such as “robust biomarker” or “strong predictor” need to be supported by areas under the receiver operating characteristic curves and confidence intervals in the external validation on independent patient cohorts [27].

This reshapes how we read scientific literature—and how cautiously and precisely it has to be written.

Authors think their arguments are clear—but this is seldom the case when a manuscript is put to peer review. Clarity is not style; it is logical “architecture,” and is improved by articulating causal structure, by the explicit explanation of assumptions, pathways, and limitations. Exposure to dense, long, ambiguous manuscripts develops the ability to identify missing conceptual links, narrative leaps, and recognize situations when figures/tables/diagrams… substitute articulate reasoning, steps in the explanation.

For instance, a manuscript describing marrow apparent diffusion coefficient–fracture associations may include flawless diffusion maps but fail to explain why other causes for edema, reconversion, or infiltration were not modeled.

### Peer review and social dynamics

Through peer review, one can come to see social projections of science: how reputation influences interpretation, how novelty can be privileged over features. Certain modalities, endpoints, or analytical frameworks dominate submission topics, while null results or negative validations struggle to surface.

Observing these dynamics cultivates a critical view on how innovation is curated/modulated by publication, and may help to advocate for methodological diversity. Awareness of this may make oneself a better author—a more realistic, perhaps less naive one—and a more ethical reviewer.

The strongest manuscripts are not flawless; they are only “defensible.” Robustness is not the absence of criticisms, but the capacity to respond coherently to those criticisms, to questioning—the capacity of a structured, solid rebuttal if needed.

Rigorous authors anticipate critique: including sensitivity analyses, external validations, and transparent reporting of uncertainty [29]. Over time, this realization reframes scientific writing, and with the experience of peer reviewing, one can learn to construct studies that would withstand interrogation.

### While reviewing, learning

Ultimately, peer reviewing is not about enforcing conformity. This section argues that it is a process of developing and polishing scientific reasoning—a true learning process, an effective way to internalize the scientific method. It helps to not accept things at “face value” when read, but to question their validity. This is particularly relevant in the current climate of rapid development of artificial intelligence (AI), in which the line with reality is blurring and it is difficult to trace sources.

Peer reviewing is modern Socratic maieutics in practice; each manuscript is an invitation to ask a number of questions: What is really being claimed…? What must be true for this to hold…? What would make this false…? Disciplined skepticism is not cynicism; it is the practical embodiment of scientific integrity. We do not become better scientists by publishing more papers. We can become better scientists by reviewing manuscripts—because it teaches us to see science not as a product, as output… but as a disciplined process of questioning. Every review is not merely a judgment of a manuscript: it refines methodological literacy, exposes hidden bias, and cultivates the habits of defensible inquiry.

Reading other reviewers’ reports is one of the most underestimated learning opportunities in academic radiology. It is the chance, the window to observe how other experts think. It is not simply about agreement or disagreement with a verdict, but about learning the craft of scientific analysis.

First of all, one learns from the things that may have gone undetected by oneself.

When several reviewers examine the same manuscript, the diversity of what they notice is quite often relevant. One may focus on acquisition parameters, another on statistical modeling, another on clinical translatability. In musculoskeletal radiology, this is particularly evident: a physicist-reviewer might critique fat/water separation modeling, while a clinician will question outcome relevance [30]. Reading these parallel interpretations teaches us that everything is seen through disciplinary lenses.

Other reviewers’ comments reveal how deep domain knowledge is operationalized. For example, a reviewer who is an expert on a particular topic may make remarks that come from experience insights, which add to our learning [31].

This ties in with the previous point—learning from the different perspective of other disciplines what has to be considered for a detailed analysis. Unfortunately, some reviews are purely censorial; others are transformative. It is very interesting to read other detailed, constructively structured reviewers’ reports, and polish how to challenge with no discouragement; how to express concerns in a way that leads to improvement rather than defensiveness [32]. This skill is not trivial—it shapes whether authors refine their science and there is true improvement of the manuscript as a result or just comply with requests automatically to be published. This is ultimately what raises quality.

The definition of rigor varies greatly among reviewers; it is difficult to make it exact to what it means to people. One reviewer may insist on external validation, another on design and aims, another on methods [33]. Seeing these expectations side by side teaches us that rigor is not abstract and it works in integration—it is achieved through different concrete demands. Over time, these standards reshape how we work on our own research and clinical practice.

It is obvious that it is very important to become aware of what one has missed, but equally instructive is recognizing what other reviewers do not notice. Occasionally, critical flaws may go unnoticed, and minor stylistic issues dominate the review report. This teaches humility: peer review is a collective process including reviewers, authors and editors, not the job of an individual reviewer.

Some reviewers privilege innovation; others reproducibility. Some reward technical novelty, others clinical applicability [34]. Reading their reports reveals that science is not governed by a single hierarchy of values. Understanding this plurality makes us more reflective authors and can make us fairer reviewers.

Finally, other reviewers’ reports make visible the social nature of scientific truth. What survives review is not only what is objectively correct, but what is intelligible, defensible, and relevant. This does not diminish science—it gives an insight into how it functions. Reading other reviewers’ comments can be a silent masterclass in scientific reasoning. It teaches not only how to judge manuscripts, but how knowledge is collectively shaped and refined.

### Peer review from the perspective of readers

From the perspective of the non-publishing, non-reviewing but scientifically engaged professional community, peer review is primarily regarded as a mechanism of quality assurance and trust-building within the scientific literature. Readers rely on peer review to filter research outputs and ensure basic methodological rigor, interpretative coherence, and relevance prior to dissemination. In radiology, where published evidence can directly influence diagnostic strategies and workflow, imaging protocols, and patient management and preferences, this role is particularly important [21, 24].

At the same time, readers are increasingly aware of the limitations and opacity of peer review, including publication delays, inter-reviewer variability, and occasional failures to detect flawed or irreproducible research [21, 35].

As a result, peer review may be viewed by readers less as a final endorsement and more as an initial checkpoint in an ongoing process that includes post-publication critique, replication, and real-world clinical validation [6, 24].

### Peer review and editorial decision-making

Peer reviews play a central but advisory role in editorial decision-making. Editors use reviewers’ evaluations to assess methodological quality, originality, ethical considerations, and clinical or scientific impact. Reviewer feedback concerning particular imaging methodology, appropriateness of technical parameters, statistical power (was the sample size preliminarily estimated?), data presentation and statistical analysis, as well as consistency with current standards of practice, may be very relevant to the editor [6, 35].

However, editorial decisions are not a mechanical reflection of reviewer recommendations. Empirical evidence shows that reviewer recommendations are strongly associated with acceptance or rejection outcomes; nevertheless, editors often assert independent judgment, especially in cases of conflicting reviews or when a manuscript is deemed strategically important for the journal and its disciplinary community [35, 36]. Editors must contextualize critiques, distinguish remediable flaws from fundamental limitations, but also consider the journal’s scope and readership.

Thus, peer reviews inform but do not dictate editorial decisions; they function as expert inputs within a broader editorial synthesis rather than as binding verdicts [6, 36].

### The dark side of peer review

Peer review is the best system we have for selecting and evaluating what a journal publishes, but it is far from perfect. For all its educational and scientific value, it carries costs and compromises and has structural weaknesses.

High-quality reviewing is intellectually demanding. It obviously competes with clinical workload, research, teaching… and eventually personal time and interests. Over time, this can lead to superficial reviews, reviewer fatigue, or declining willingness to engage deeply—particularly in radiology, where manuscripts are increasingly technical and data-heavy.

In addition, reviewers are experts in what already exists. This makes the system inherently conservative. Truly disruptive ideas—novel biomarkers, unconventional modeling approaches, atypical imaging paradigms—may be judged as “insufficiently validated” not because they are flawed, but because they fall outside existing frameworks, current knowledge does not reach [37]. Peer review can therefore act as a stabilizer of the status quo and end up hindering knowledge, instead of advancing it.

Besides, two equally qualified reviewers may reach entirely different conclusions about the same manuscript. What one sees as a fatal bias, another views as an acceptable limitation in a context [38]. This variability reflects the plurality of scientific values—but it also means that publication decisions can be influenced as much by who reviews the paper as by the quality of the paper itself.

Anonymity protects reviewers, but it can also favor unconscious bias—against institutions, world regions, methods, or even certain medical or imaging subspecialties. Junior authors or unconventional groups may face a higher barrier to acceptance, not through overt discrimination, but through differential expectations [39].

In fast-moving fields such as imaging AI or advanced quantitative MRI, prolonged review cycles can make findings obsolete before they appear. The balance between rigor and timeliness is difficult to maintain, as the complexity of the reviewing process increases.

For authors, harsh or poorly framed reviews can be demoralizing. For reviewers, repeated exposure to low-quality work can be boring and generate cynicism. Without a culture of constructive critique, peer review risks becoming corrosive rather than educational.

Peer review remains indispensable, but it is not neutral. It shapes what science becomes—not only by what it accepts, but by what it discourages, delays, or exhausts. Recognizing its limitations is not a rejection of the system, but a prerequisite for making it wiser, fairer, and more sustainable.

This “dark side” of peer review—including bias, lack of transparency, inconsistency, and occasionally unconstructive or unethical reviewer behavior—has been extensively discussed in the literature [21, 24]. Several strategies have been proposed to mitigate these limitations.

Increased transparency, such as open peer review with publication of reviewer reports or disclosure of reviewer identities, has been shown to improve accountability and tone, although it may affect reviewer willingness in some contexts [23]. Structured review templates and reviewer training can reduce variability and enhance the consistency of evaluations [6, 24]. This is particularly relevant in technically complex fields within radiology, such as quantitative imaging or machine learning. In papers using AI methods, the reporting of external validation, calibration, data leakage, reference standard circularity, and availability of code/model documentation needs to be assessed for completeness [40–46].

Additionally, diversification of reviewer pools across geography, gender, career stage, and methodological expertise can help address systemic biases [24]. Strong editorial oversight, including moderation of inappropriate reviews and active mediation between authors and reviewers, remains essential [36]. Finally, post-publication commentary and data-sharing initiatives support a shift from peer review as a single pre-publication event to a continuous evaluative process [6, 23].

While no peer-review model is free of limitations, these measures can substantially reduce its opacity and improve fairness, rigor, and trustworthiness.

## Conclusions

A fitting reflection may be drawn again from Seneca, who reminded his pupils that *“non scholae sed vitae discimus”*—we learn not for school, but for life (*Epistulae Morales ad Lucilium*, 106.12) [11].

Peer review is not an academic ritual performed for journals, but a disciplined exercise in intellectual character, shaping how we reason, doubt, and defend knowledge. Each manuscript we review refines not only the science before us, but the scientist within us.

In this sense, peer reviewing is less an obligation than a lifelong training in epistemic virtue—a quiet but enduring apprenticeship in how radiology, and science itself, ought to think.
